# Insulinoma Misdiagnosed as Post-bariatric Hypoglycemia: A Case Report and Review of the Literature

**DOI:** 10.7759/cureus.38197

**Published:** 2023-04-27

**Authors:** Emre Teke, Yasin Güneş, Mehmet T Aydın, Eylem Cagiltay, Seda Sancak

**Affiliations:** 1 General Surgery, Haydarpasa Numune Training and Research Hospital, istanbul, TUR; 2 General Surgery, Fatih Sultan Mehmet Training and Research Hospital, Istanbul, TUR; 3 Endocrinology and Metabolic Diseases, University of Health Sciences, Sultan Abdulhamid Han Education and Research Hospital, Istanbul, TUR; 4 Endocrinology and Metabolic Diseases, Fatih Sultan Mehmet Training and Research Hospital, Istanbul, TUR

**Keywords:** hypoglycemia, diabetes mellitus, bariatric surgery, insulinoma, post-bariatric hypoglycemia

## Abstract

Hypoglycemia is seen with increasing frequency after bariatric surgery. After the diagnosis of hypoglycemia has been clarified, malnutrition, drugs, hormone deficiencies, insulinoma, extra-islet tumors, post-bariatric hypoglycemia (PBH), early or late dumping syndrome, and nesidioblastosis should be considered in the differential diagnosis. A few case reports of insulinomas presenting after bariatric surgery have been reported in the literature. The coexistence of insulinoma and type 2 diabetes mellitus (T2D) is very rare. We herein report a clinical case of insulinoma presenting with severe hypoglycemia in a patient with a history of gastric transit bipartition.

A patient with type 2 diabetes mellitus underwent gastric transit bipartition surgery due to the inability of medical therapy to provide adequate hyperglycemia control. After the operation, hypoglycemic symptoms appeared, and a reversal operation was performed, considering the diagnosis as PBH. After the reverse operation, the patient's hypoglycemia symptoms did not regress. The patient was admitted to our endocrinology clinic due to the persistence of hypoglycemia and symptoms such as fatigue, palpitation, and syncope. The patient's detailed anamnesis was examined, additional tests were performed, and the patient was diagnosed with insulinoma. The symptoms of hypoglycemia and the need for treatment for diabetes mellitus disappeared after the Whipple operation.

This is the first case of insulinoma after gastric transit bipartition and subsequent reversal operations. In addition, the patient's diagnosis of diabetes mellitus makes this case unique. Although this is a very rare case, clinicians must be aware of it, especially if the patient has hypoglycemic symptoms during the fasting state.

## Introduction

The management of obesity consists of lifestyle alterations, medical therapy, and surgical interventions. Bariatric surgery (BS) has recently been accepted as the most effective and durable option, especially in cases of morbid obesity and glucose metabolism disorders [1]. Obesity surgery offers substantial improvements in glucose metabolism postoperatively, is more successful than medical therapies at enhancing type 2 diabetes (T2D) management, and decreases the requirement for drugs [2].

In addition to all of these metabolic advantages, there is an increased prevalence of hypoglycemia. Hypoglycemia developing after bariatric surgical procedures is a complication with increasing frequency. The incidence of post-surgical hypoglycemia is unclear due to differences in the diagnostic criteria for hypoglycemia and often nonspecific symptoms. After the diagnosis of hypoglycemia has been clarified, malnutrition, drugs, hormone deficiencies, insulinoma, extra-islet tumors, post-bariatric hypoglycemia (PBH), early or late dumping syndromes, and nesidioblastosis should be considered in the differential diagnosis [2,3]. We herein report a clinical case of insulinoma presenting with severe hypoglycemia in a patient with a history of gastric transit bipartition and T2D.

## Case presentation

A 62-year-old female patient was referred to our hospital with a preliminary diagnosis of insulinoma. She was hypothyroid due to a total bilateral thyroidectomy and had an atrial fibrillation diagnosis. In 2005, T2D was diagnosed with a hemoglobin A1c (HbA1c) of 8% and was first treated with metformin and then with insulin.

She had a gastric transit bipartition operation because medical treatment could not provide adequate hyperglycemia control. Prior to her BS, laboratory findings confirmed that her HbA1c level was 8.4%, her fasting glucose level was 165 mg/dL, and her BMI was 41.5 kg/m2. During the procedure, all hypoglycemic agents were stopped. On the seventh postoperative day, the patient began to exhibit frequent, mostly fasting, hypoglycemia in the range of 40-45 mg/dL. After the patient's complaints regressed, she was discharged. Since she had hypoglycemia complaints again in the fourth month of her discharge, diet treatment was given, considering late dumping syndrome. The patient continued to exhibit hypoglycemia symptoms despite all dietary modifications. After hypoglycemia symptoms did not resolve, a reversal operation was planned, considering PBH.

Six months after the first operation, it was observed that the ileal loop from the gastrojejunostomy anastomosis to the jejunojejunostomy anastomosis formed a cocoon. This loop was removed, and the patient regained her normal anatomy. No postoperative complication was detected, and the patient was discharged on the seventh postoperative day. The patient was admitted to our endocrinology clinic nine months after the first operation due to the persistence of hypoglycemia and symptoms such as fatigue, palpitation, and syncope.

When the patient's thorough anamnesis was analyzed, it was determined that the patient had a sense of weakness and faintness one to two hours after eating and that palpitations, perioral numbness, diaphoresis, disorientation, and syncope occurred at night. She said that she got up at night and ate because of her symptoms, and her symptoms were completely relieved by oral glucose. Initially, she was able to control her hypoglycemia symptoms with frequent meals; however, her symptoms worsened so much that she had to make many trips to the emergency department due to low glucose levels in the 30 mg/dl range. Her current BMI was 32.8 kg/m2, and her HbA1c level was 6.1%. The patient initially lost 36 kg following her BS.

When there was no response to dietary advice, hyperinsulinemia was investigated using biochemical testing. Blood tests measured at the episode yielded hypoglycemia (35 mg/dL) without suppression of IRI (25.5 μU/mL) or C-peptide (5.51 ng/mL) levels.

In addition, when the patient's previous analyses were examined. Abdominal multiphase computed tomography (CT) taken one month before the first operation revealed, incidentally, a benign mature solid lesion of 15 mm in the head of the pancreas. At that time, this lesion was thought to be a neuroendocrine tumor. Since the lesion size was less than 2 cm, it was found to be coincidental, and the patient had no complaints; she was followed up.

**Figure 1:**
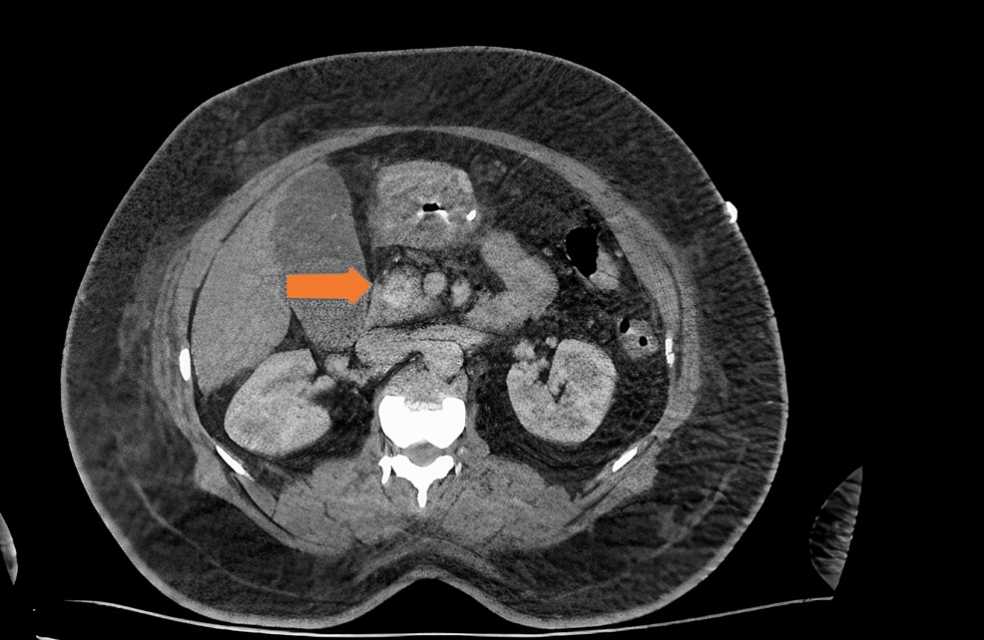
A CT of the abdomen showed a solitary nodule in the pancreas.

Computed tomography (CT) and positron emission tomography-computed tomography (PET CT) scans were performed. On the CT of the upper abdomen with IV contrast, in the head of the pancreas, a hyperdense mass lesion of approximately 15x12 mm in size compared to the lobulated contoured pancreatic parenchyma with relatively smooth borders was noted (Figure 1). The mass was in the head of the pancreas and adjacent to the pancreatic duct. The Whipple procedure was applied because the localization of the mass was not suitable for enucleation.

Postoperative specimen pathology detected a well-differentiated neuroendocrine tumor. The sample was positive for synaptophysin, chromogranin, CD56, CD57, neuron-specific enolase (NSE), inhibin, and B-catenin. Ki-67 was 2%. The patient was discharged on the tenth postoperative day. The patient did not need to use insulin during the three-month follow-up. There were no hypoglycemic symptoms.

## Discussion

Post-bariatric hypoglycemia is a complication of BS whose prevalence and associated risk factors are incompletely understood. Hypoglycemia is often asymptomatic after upper gastrointestinal system surgery [3]. Less than 1% of patients have severe hypoglycemia that requires hospitalization; however, estimates imply that symptomatic hypoglycemia occurs in 10%-30% of individuals and occurs following both Roux-en-Y gastric bypass (RYGB) and vertical sleeve gastrectomy (SG). However, it has been reported following other procedures, such as the duodenal switch, in which nutrients are supplied straight to the middle or end of the small intestine [4]. The possible causes of hypoglycemia after BS include post-bariatric hypoglycemia (PBH), early or late dumping syndromes, nesidioblastosis, and rarely, insulinoma [3,5].

Hypoglycemic symptoms in patients with BS are often associated with dumping syndrome [6]. PBH is difficult to distinguish from late dumping syndrome because nonspecific symptoms are considered dumping syndrome and can therefore be difficult to diagnose [7].

After a 12-hour fast, glucose, insulin, C-peptide, proinsulin, beta-hydroxybutyrate, and cortisol levels should be measured in patients with chronic hypoglycemia episodes in order to rule out an autonomous source of insulin production. In a spontaneous episode or one induced by a 72-hour fast (fasting hypoglycemia) or a mixed-meal test (postprandial hypoglycemia), a biochemical analysis may be conducted [2]. Insulinoma should be investigated if independent insulin secretion is detected. Hypoglycemia when fasting is the primary symptom of insulinoma. It is difficult to differentiate insulinoma from reactive hypoglycemia if insulinoma patients mostly display postprandial hypoglycemia [2].

As seen in our patient, it is essential that hypoglycemia occurring predominately in the fasting state, occurring more often at night, worsening early after BS, and being resistant to diet after BS be studied further to rule out insulinoma. Moreover, patients with diabetes who have undergone BS are less likely to develop hypoglycemia. The possible reason for this is the absence of increased β-cell function and decreased insulin resistance, which are necessary for the development of hyperinsulinemic hypoglycemia in diabetic patients. In addition, diabetic patients are less sensitive to the effects of Glucagon-like peptide-1 (GLP-1) agonists. Therefore, the development of hypoglycemia in diabetic patients takes longer than in non-diabetic patients [8]. Therefore, a rare insulinoma should be excluded in patients, especially if fasting hypoglycemia occurs early after BS or shows other atypical features [8]. The last stage is to identify the tumor's anatomical location and rule out the presence of any other tumors. In our situation, a CT scan enabled the identification of an insulinoma. The definitive treatment for insulinoma comprises complete surgical resection. When the mass is small, benign, solitary, and superficial and the pancreatic duct is not involved, the best surgical approach is laparoscopic enucleation [8]. In our case, the mass was in the head of the pancreas and adjacent to the pancreatic duct. Therefore, the Whipple procedure was preferred instead of enucleation.

It is conceivable that our patient's prior history of type 2 diabetes might have masked her symptoms. Our patient was using insulin before the operation and did not describe symptoms of hypoglycemia. After she lost weight, she was no longer "protected" by insulin resistance, which was likely a result of her obesity-induced resistance to the extra insulin her tumor was producing [9].

In addition, the postoperative hormonal environment may have contributed to increased β-cell proliferation in susceptible individuals after gastric surgery, which may have led to the emergence of insulinoma. The removal of the insulinoma normalized her blood sugar levels.

## Conclusions

This is the first case of insulinoma after gastric transit bipartition and subsequent reversal operations. In addition, the patient's diagnosis of diabetes mellitus makes this case unique. Bariatric surgeons should be aware of metabolic symptoms, including hypoglycemia, that develop after bariatric surgery. Before bariatric surgery, a multidisciplinary approach should include careful clinical assessment.
